# The Use of Routine Laboratory 17‐Hydroxyprogesterone for Identification of Cases of 21‐Hydroxylase Deficiency Congenital Adrenal Hyperplasia

**DOI:** 10.1111/cen.70058

**Published:** 2025-11-21

**Authors:** Joseph McElvaney, Salma R. Ali, Amy R. Frank, Sophie Longmuir, Jane McNeilly, Malika Alimussina, Ruth McGowan, Syed Faisal Ahmed

**Affiliations:** ^1^ Developmental Endocrinology Research Group, Royal Hospital for Children University of Glasgow Glasgow UK; ^2^ Department of Clinical Biochemistry Glasgow Royal Infirmary Glasgow UK; ^3^ West of Scotland Centre for Genomic Medicine Queen Elizabeth University Hospital Glasgow UK; ^4^ Department of Clinical Biochemistry Royal Hospital for Children Glasgow UK

**Keywords:** anthropometry, blood pressure, BMI, CAH, incidence

## Abstract

**Background:**

Clinical outcome studies of 21‐hydroxylase deficiency congenital adrenal hyperplasia (21OHD CAH) may be subject to selection bias due to incomplete case ascertainment. This study aimed to develop a methodology for identifying existing CAH cases and explore its utility to study clinical outcomes.

**Methods:**

17‐hydroxyprogesterone assays (17OHP) processed in NHS Greater Glasgow and Clyde between 2014 and 2022 were analysed based on 17OHP result (≥ 6 or < 6nmol/L), location, test frequency and clinical details. Identified cases were cross‐referenced against local clinical data logs. For confirmed cases, current age, sex, age at diagnosis, mortality status, most recent blood pressure (BP) and anthropometry were collected.

**Results:**

Assay results from 57,011 cases were extracted and, of these, 116 (F:M, 81:35) had confirmed CAH but 66 (57%) were not reported by any local clinical data logs. The median age at the time of the study was 33 years (range, 2, 75) and 95 (82%) were over 16 years (F:M, 69:26). In these adults, 52 (55%) were diagnosed in childhood (i.e. ≤ 16 years) and only 1 male was diagnosed in adulthood. The median body mass index (BMI) standard deviation score of children was 0.70 (−2.43, 3.15). Median BMI of adults was 28 (15, 56) and median adult and paediatric systolic BP was 120 mmHg (95, 153) and 106 mmHg (83, 130), respectively.

**Conclusion:**

The 17OHP‐based algorithm that was used in this study represents a useful method for identifying existing cases of CAH and can allow improved understanding of routinely collected markers of clinical outcome.

## 1 Introduction

Congenital adrenal hyperplasia (CAH) due to 21‐hydroxylase deficiency (21OHD) is the most common genetic cause of primary adrenal insufficiency and can have a wide range of clinical manifestations depending on the extent of enzymatic deficiency [1]. When it presents in infancy, the phenotypic manifestations are usually secondary to a combination of cortisol and aldosterone deficiency and androgen excess. 21OHD CAH can also present later in childhood with premature virilisation in boys and girls without any evidence of salt‐losing, often referred to as simple virilizing CAH [2]. The incidence of these classical forms of 21OHD CAH is reported to be around 1 in 14,000–18,000 births [3]. 21OHD CAH can also present in adolescence or adulthood, when it is often referred to as non‐classical CAH, and may be the most common monogenic endocrine disorder with a prevalence of 0.5% in Caucasians and a prevalence of 4%–5% in women with signs of androgen excess [4, 5]. The diagnosis of 21OHD CAH is usually based on a raised serum 17‐hydroxyprogesterone (17OHP), the cornerstone investigation which is followed by other biochemical and genetic confirmatory tests. Cases of the classical forms of CAH usually receive lifelong specialist care with regular assessment of the steroid metabolites, including 17OHP, but the management of cases of non‐classic CAH may be more variable [1]. Furthermore, knowledge of long‐term outcomes in CAH is scarce partly due to a lack of studies that systematically monitor outcomes, as well as incomplete ascertainment of cases that have reached adulthood [6, 7]. Although regions that have newborn screening programmes may be able to identify all childhood cases within a specific regional or national programme, this may be challenging in regions where a screening programme does not exist and when care may be delivered through networks of clinics [8]. Irrespective of whether a screening programme exists or not, the identification of adult cases of CAH may be even more challenging, given that most screening programmes have not existed for longer than 2–3 decades [9]. The objective of the current study was to understand the prevalence of 21OHD CAH within a specific UK health care region, NHS Greater Glasgow and Clyde Health Board (NHS GGC), using the records in the regional biochemistry laboratory that performs the 17OHP assay for the whole region. This would not only allow an improved understanding of the prevalence of 21OHD CAH but it would also lay down the foundation for performing similar exercises in other comparable regions to improve CAH case ascertainment for long‐term outcome studies.

## 2 Materials and Methods

### 2.1 Case Identification

Details of all cases for whom a serum 17OHP had been processed by NHS GGC between 2014 and 2022 were extracted from the clinical biochemistry laboratory information system. These details included the community health index (a unique identifier that is assigned in Scotland to all individuals requiring health care), the date of birth, sex, location of the health care facility from which the sample was requested, any clinical details provided by the requesting clinician and the results of the 17OHP assay. Given that some cases of CAH may have their care shared between NHS GGC and other health care regions, and the regional biochemistry laboratory may also serve other health care regions, the test request location was categorised as either solely within NHS GGC, secondary care hospitals in neighbouring health boards or outside of NHS GGC catchment. This latter group was excluded from the analysis. The study was performed as part of health care service evaluation and development within NHS GGC and did not require NHS research ethics committee review [10].

**Figure 1:**
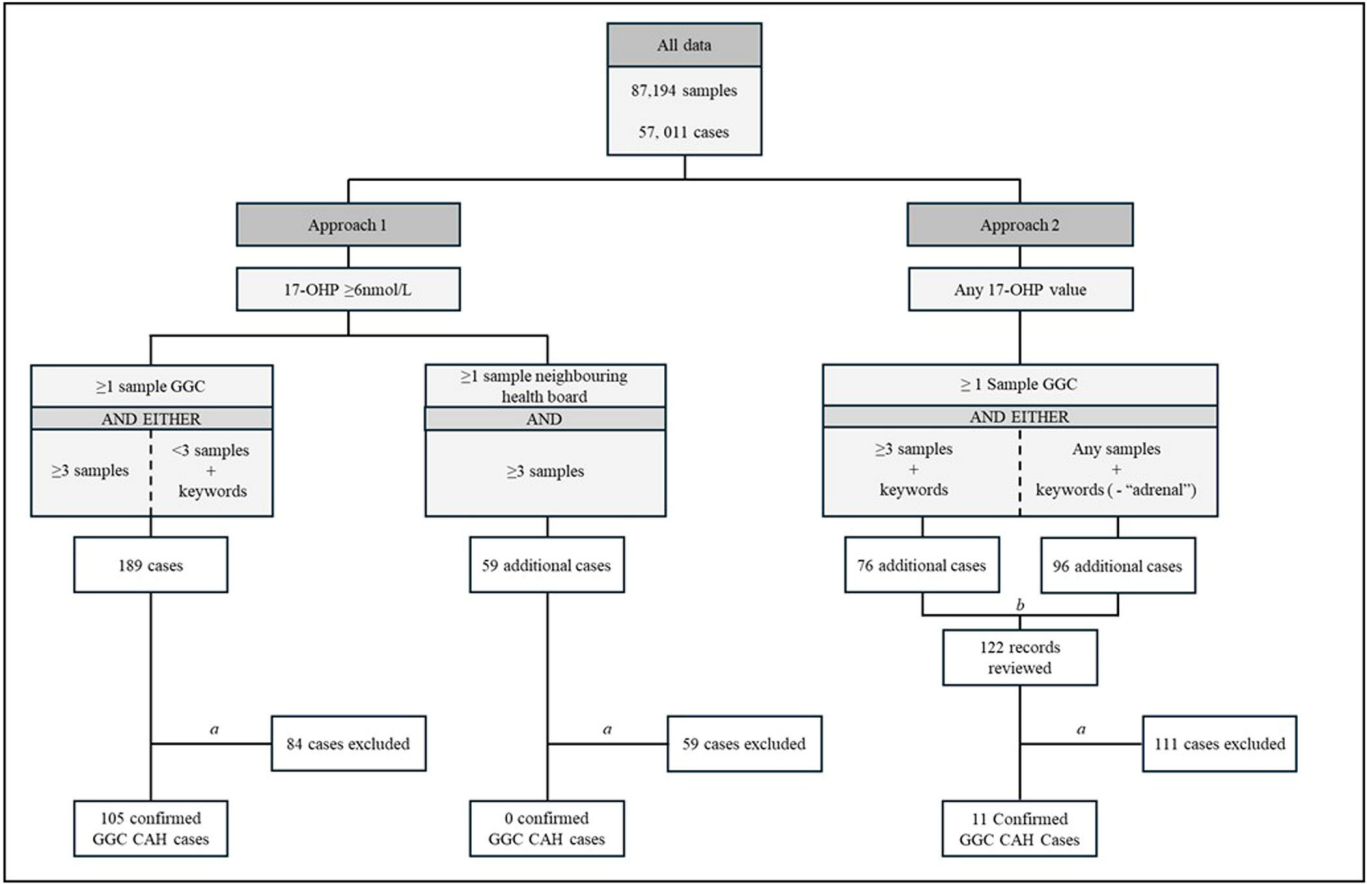
Case identification algorithm comprising 2 approaches. Approach 1 examined cases with 17OHP value of ≥ 6 nmol/L. Cases with at least 1 sample requested within NHS GGC were reviewed if they had at least three samples *or* one or two samples but request clinical details included keywords (below). Cases with at least one sample requested in a neighbouring health board were reviewed if they had at least three samples. Approach 2 examined all cases irrespective of 17OHP value, if they had at least 1 sample requested in NHS GGC. Cases were reviewed if they had at least three samples and keywords *or* one or two samples and keywords except ‘Adrenal’. Keywords: ‘CAH’, ‘Congenital’, ‘Hyperplasia’, ‘Hypoplasia’, ‘Salt’, ‘Wasting’, ‘Classical’, ‘Non‐classical’ or ‘Adrenal’. a Excluded as no diagnosis of 21OHD and/or care outside of GGC: b 122 records reviewed in Approach 2 due to overlap in cases identified. 17‐OHP, 17‐hydroxyprogesterone; CAH, congenital adrenal hyperplasia; GGC, NHS greater glasgow and clyde.

Identification of cases was performed in two approaches (Figure 1). A 17OHP value of ≥ 6 nmol/L was determined as a threshold based on the reference range for the liquid chromatography‐tandem mass spectrometry assay at the regional laboratory where the assays were performed. In Approach 1, cases with three or more samples showing a 17OHP value of ≥ 6 nmol/L with at least one requested from NHS GGC or a neighbouring health board were selected for a review of the clinical records. Those cases with one or two samples with a 17OHP value of ≥ 6 nmol/L and at least 1 requested in NHS GGC were also included if the test request included the keywords: ‘CAH’, ‘Congenital’, ‘Hyperplasia’, ‘Hypoplasia’, ‘Salt’, ‘Wasting’, ‘Classical’, ‘Non‐classical’ or ‘Adrenal’. In Approach 2, further cases were identified for review of clinical records by examining those that had any 17OHP result, three or more samples and the above keywords. Cases were also included irrespective of the number of samples and 17OHP result, as long as they had the relevant keywords (excluding ‘Adrenal’) in the test request.

### 2.2 Case Confirmation and Collection of Clinical Data

Once identified for review, electronic health records in NHS GGC were used to confirm cases of 21OHD CAH through clinical correspondence, clinical coding and genetics when available. Databases held by clinical services were also used to cross reference the cases of CAH identified by using 17OHP data. These comprised paediatric clinic data, cases with CAH who had undergone urine steroid analysis and genetically confirmed cases of 21OHD. For confirmed cases of CAH, clinical characteristics that were collected included age at time of study, age at diagnosis (≤ 16 years or > 16 years), responsible consultant, elapsed time since last consultation, mortality status, most recent anthropometry and systolic/diastolic blood pressure (SBP/DBP).

### 2.3 Data Analysis

All data were described as medians and ranges. Sex at birth was used throughout. Anthropometric data were converted to standard deviation scores (SDS) to correct for age and sex using 1990 reference data for Great Britain and Northern Ireland [11]. SDS were generated using Growth Analyser RCT software, V4.2.12, Rotterdam, the Netherlands. For adults, if age at height measurement was greater than the limit of the Growth Analyser reference data (i.e., 23 years old), SDS were generated for this age. World Health Organisation‐defined body mass index (BMI) cut‐offs were used to classify cases as ‘overweight’ and ‘obese’ [12]. For cases aged 4–16 years an average of the 98th centile for boys and girls living in the United Kingdom was used as a blood pressure theshold [13]. For adults, National Institute for Health and Care Excellence recommendations for suspecting hypertension were used [14]. Analysis was conducted using Microsoft Excel for Microsoft 365 Version 2503 [15]. Where sub‐groups were compared, Mann–Whitney *U* test was calculated using RStudio version 4.4.2 and *p* < 0.05 was considered significant.

## 3 Results

### 3.1 Case Identification

Biochemistry data extraction included a total of 87,194 17OHP assay results from 57,011 cases (Figure 1). In Approach 1, 248 cases were identified for clinical record review with 105 cases of CAH identified (42%). No CAH cases under the care of GGC clinician were identified where samples were solely requested from a neighbouring health board. A further 122 records were reviewed in Approach 2 analysis, identifying 11 additional cases of CAH (9%). A total cohort of 116 cases were therefore identified, with 91% through Approach 1. A total of 33 cases were reported in the diagnostic genetic laboratory service database and 32 of these were identified through the search algorithm employed above. The additional single case had been diagnosed after the study period. Similarly, 32 cases were reported by the steroid biochemistry laboratory service records and all of these cases were also identified by the algorithm. Lastly, 25 cases were reported by the paediatric endocrine service database and all these cases were also identified by the algorithm. Of the 116 cases of 21OHD cases identified by the algorithm, 66 (57%) cases were not reported by any locally‐held clinical service database.

### 3.2 Clinical Description

At the time of the study, the median age of the 116 cases (F:M, 81:35) was 33 years (range 2, 75) with 95 (82%) cases (F:M, 69:26) being over 16 years old and 21 (22%) over 50 years. For these adults, 52 (55%) (F:M, 31:21) were diagnosed at an age of 16 years or under and 37 (39%) (F:M, 36:1) were diagnosed after the age of 16 years; in the remaining 6 (6%) the age at diagnosis was not clear. For adults, median time since last consultation was 10 months (range, 1, 108) and 69 (73%) had been seen within the previous 18 months. For the 21 cases (F:M, 12:9) that were 16 years or under, median time since last consultation was 9 months (3, 105) and 15 (71%) had been seen within 18 months. Of the 116, 113 (97%) of the cohort were alive at the time of study.

### 3.3 Outcomes in Children

**Figure 2:**
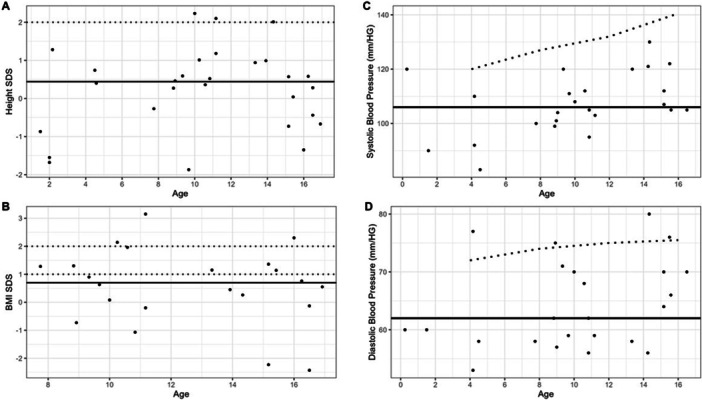
Anthropometric and blood pressure outcomes at latest encounter in children and adolescents (i.e., at the age of 16 years or less) with CAH. Horizontal black line indicates the median. (A) Height SDS for measurements in childhood with dashed line at 2 SDS; (B) BMI SDS for measurements at age 5–16 years old with dashed lines for overweight and obese categories as defined by WHO [12]; (C) systolic blood pressure with dashed line at 98th centile [13]; (D) diastolic blood pressure with dashed line at 98th centile [13]. BMI, body mass index; CAH, congenital adrenal hyperplasia; SDS, standard deviation score; WHO, World Health Organisation.

There were a total of 28 cases in whom the most recent anthropometric data were available at an age under 16 years old. This included the 21 cases that were less than 16 years old and an additional 7 cases who were over the age of 16 years at the time of study. For these 28 cases, at a median age of 11 years (1, 16), the median weight SDS was 0.83 (−1.96, 3.64) and median height SDS was 0.44 (−1.87, 2.23) (Figure 2). For the 22 cases aged between 5 and 16 years at the time of measurement, median BMI SDS was 0.70 (−2.43, 3.15) (Figure 2). Of these, 6 (27%) were overweight and 3 (14%) were obese. Median SBP and DBP were 106 mmHg (83, 130) and 62 mmHg (53, 80) respectively for the 24 cases with blood pressure measurements available in childhood. Of the 21 cases aged 4–16 years at measurement, no children had an SBP and 4 (19%) had a DBP > 98th centile.

### 3.4 Outcomes in Adults

**Figure 3:**
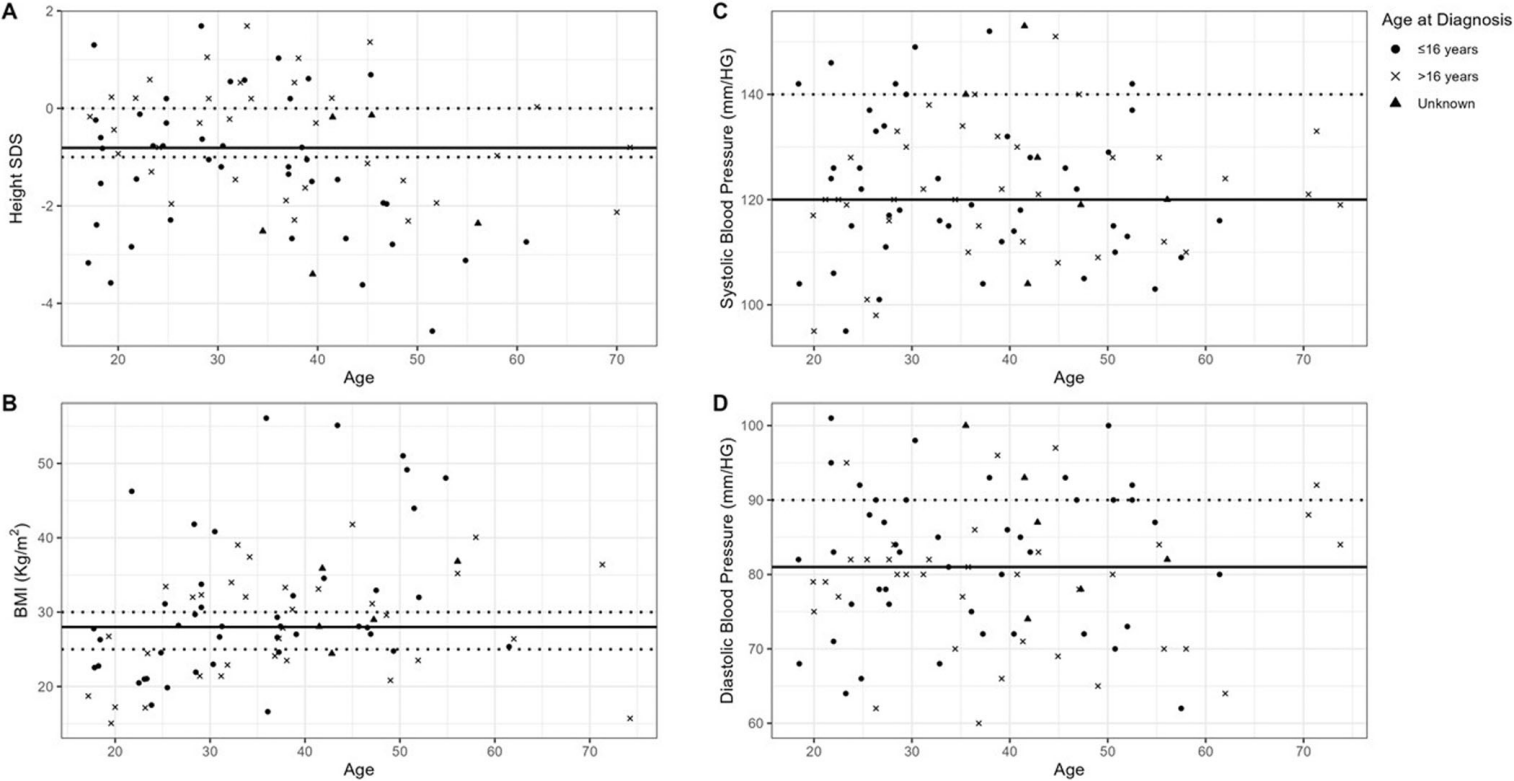
Anthropometric and blood pressure outcomes at latest encounter in adults with CAH. Horizontal black line indicates the median. (A) Height SDS for measurements at final adult height with dashed lines at 0 SDS and −1 SDS; (B) BMI measurements in adulthood with dashed lines for overweight and obese categories as defined by WHO [12]; (C) Systolic blood pressure with dashed line for hypertensive range; (D) Diastolic blood pressure with dashed line for hypertensive range. BMI, body mass index; CAH, congenital adrenal hyperplasia; SDS, standard deviation score; WHO, World Health Organisation.

Of the 95 adults, most recent height was available in 80 adults, recorded at a median age of 34 years (17, 71). Median height SDS was −0.81 (−4.57, 1.69) with 73% having an SDS < 0 and 46% having SDS ≤ −1. Most recent height was available for 60 women with a median SDS of −0.54 (−3.62, 1.69). Age at diagnosis was known for 75 cases (F:M 58:17) with most recent height available. For the 26 (43%) women diagnosed at 16 years or under, median height SDS was −0.93 (−3.62, 1.69), compared to −0.30 (−2.31, 1.69) for the 32 (53%) women diagnosed over 16 years (*p* = 0.24) and −1.28 (−4.57, 0.55) for the 16 males diagnosed 16 years or under (*p *= 0.17). Most recent BMI acquired in adulthood was available in 81 (85%). The median BMI of these cases was 28 (15, 56). Of these, 21 (26%) were overweight and 33 (41%) were obese (Figure 3). For those with available data and known age of diagnosis, the median BMI of the 43 diagnosed at 16 years or under was 28 (17, 56) and also 28 (15, 42) for the 33 diagnosed after the age of 16 years. Of the 95 adults, 85 (89%) had clinic blood pressure readings available and most recent median SBP and DBP of these cases were 120 mmHg (95, 153) and 81 mmHg (60, 101), respectively. Of the 85, 12 (14%) had an SBP of 140 mmHg or greater and 19 (22%) had a DBP of 90 mmHg or greater.

## 4 Discussion

Studies of long outcome in 21OHD CAH may be subject to selection bias due to incomplete case ascertainment. In regions where newborn screening is not available, identification of existing cases of 21OHD needs to be performed through alternative methods. This study developed and employed an algorithm based on 17‐OHP assay results that successfully captured a large cohort of cases with 21OHD in one health board region in the United Kingdom. The study also shows that this exercise can pave the way to effective monitoring of long‐term outcomes.

The prevalence of 21OHD CAH in newborns has previously been studied in Scotland through the analysis of neonatal blood spots and was reported to range between 3:100,000 and 8:100,000 [16]. NHS GGC covers a population of 1.2 million people and based on the 116 cases that were identified in the current exercise, that would provide a prevalence of approximately 9.7:100,000. If those who were diagnosed in adulthood are removed from the identified cases, then the prevalence of the classic forms of CAH was 6:100,000. This reported prevalence is similar to what has been reported in other populations. A recent population‐based study in Denmark which has a population of 5.9 million identified a total of 462 cases of CAH, that is, a prevalence of 7.8:100,000 [17]. While it is of course possible that the prevalence may have been higher in Scotland if there was a screening programme, the main purpose of the current study was to develop a process to identify as many cases as possible so that large scale studies of clinical outcomes can be performed through the analysis of routinely collected data. Indeed, the F:M ratio in the present study is reflective of prior cumulative prevalence studies but is divergent from those focusing on newborn screening, likely due to the inclusion of the predominantly female cases of non‐classic CAH [8]. The algorithm employed in the current study has allowed the investigators to identify the large majority of cases that are under clinical care. Of particular note, approximately half of the cases that were identified were not present in any databases held by the diagnostic or clinical services with almost all of these identified by searching for three routine laboratory 17OHP that were 6nmol/l or greater.

A previous study in 17 UK centres, the Congenital adrenal Hyperplasia Adult Study Executive (CaHASE) study, identified 373 cases of whom 199 agreed to participate, indicating a case identification rate of 22 per centre and an inclusion rate of approximately 12 per centre [18]. An international study of 13 centres participating in the I‐CAH Registry was able to report on 244 adults which is an inclusion rate of almost 19 adults per centre [6]. In contrast, in the current study, a single centre was able to identify 95 adults in its care, highlighting the level of selection bias that may exist when studying and reporting on clinical outcomes in rare conditions such as CAH. This was further illustrated by the point that the current study identified 36 female cases of CAH diagnosed in adulthood in a single centre compared to 31 female cases of non‐classic 21OHD that were included from 17 centres in the UK CaHASE study [18].

The preliminary data that was reported on clinical outcomes focussed on those aspects of clinical care that are universally considered to be routine in 21OHD CAH and included anthropometric indices and blood pressure [3, 6]. By identifying cases through the algorithm employed in this study and by aiming to report these routine clinical outcomes, the study was able to provide a meaningful and representative snapshot of clinical care. It was reassuring to observe that despite the concerns regarding case ascertainment, the basic characteristics of the previous UK‐based study, the CaHASE cohort, were comparable to the current group with respect to age and sex distribution with a median age of about 35 and preponderance of female cases.

In accordance with other reports, the study was also able to confirm the finding of a raised BMI in adults with CAH. A recent review of the cardiometabolic aspects of CAH by Krysiak and colleagues found substantial evidence of an association between CAH and overweight and obesity [19]. Of particular demographic relevance to the current study, retrospective analysis of 307 CAH cases in England reported a median BMI of 26.7 kg/m^2^ and which was comparable to the current cohort [20]. The CaHASE cohort, as well as a more recent retrospective report of 254 adults with CAH in the United States, also demonstrated an obesity rate of about 40% also aligning closely to our cohort [18, 21]. While overweight rates in the current study were high at 26%, the CaHASE group had a higher rate at 37%. Evidence regarding BMI comparison of classic and non‐classic forms of CAH is complex [19]. The CaHASE cohort, for instance, identified that adult women with classic CAH were shorter and had higher BMI than those with the non‐classic form of the disease [22]. By contrast, a larger study of 545 cases in the Swedish National CAH Register reported that non‐classic CAH cases had the greatest risk of obesity [23].

Previous studies of linear growth in CAH suggest that final adult height is frequently impaired and although infants are generally shorter in length, older children and young adolescents tend to have taller stature than their peers [24, 25]. This general trend was reflected in the current study, where height was above average in childhood whilst adults were shorter than average, a finding that was consistent with that reported in the CaHASE cohort [22]. However, in a review of 35 studies Muthusamy and colleagues reported an average height SDS of −1.4 for adults and this was lower than −0.8 which was observed in the current study [25].

Elevated SBP and DBP is frequently reported in adults with CAH and highlights the importance of BP assessment and monitoring in CAH [3, 6, 19, 26]. In the current study, almost 90% of cases had a blood pressure measurement that was available in the case records and 14% and 22% of cases had a systolic and diastolic blood pressure that was in the hypertensive range, respectively. Population studies indicate that the prevalence of hypertension is about 20%–25% of the general adult population [27, 28] and while this is comparable to the rates in the current study of adults with CAH, the findings presented here do not include cases who may have been normotensive at the time of the study while on anti‐hypertensive therapy. Furthermore, presentation of absolute blood pressure values limits interpretation in a cohort that ranges from 2 to 75 years old.

In summary, the algorithm employed in this study has assisted in selecting appropriate cases to review when identifying existing cases of CAH. Although it is possible that some cases may not have been identified, the size of our cohort aligns with previously published prevalence estimates and the method also identified all known cases that were reported by the clinical services. The outcomes that were examined focussed on those that are routinely collected and these provided a more comprehensive and contemporary snapshot of CAH care in our local region. Further analysis of this cohort could include more detailed health status assessment through standardised assessments or through linking other routinely collected data sets.
